# Global Human Threat: The Potential Synergism between Mercury Intoxication and COVID-19

**DOI:** 10.3390/ijerph20054207

**Published:** 2023-02-27

**Authors:** Gabriela de Paula Arrifano, Marcus Augusto-Oliveira, Amanda Lopes-Araújo, Letícia Santos-Sacramento, Barbarella Matos Macchi, José Luiz Martins do Nascimento, Maria Elena Crespo-Lopez

**Affiliations:** 1 Laboratory of Molecular Pharmacology, Institute of Biological Sciences, Federal University of Pará, Belém 66075-110, PA, Brazil; 2 Laboratory of Neurochemistry and Cellular Biology, Institute of Biological Sciences, Federal University of Pará, Belém 66075-110, PA, Brazil

**Keywords:** SARS-CoV-2, coronavirus, mercury, COVID-19, methylmercury, exposure, sequelae, MeHg, ASGM, gold mining, Amazon

## Abstract

The COVID-19 pandemic affected billions of people worldwide, and exposure to toxic metals has emerged as an important risk factor for COVID-19 severity. Mercury is currently ranked as the third toxic substance of global concern for human health, and its emissions to the atmosphere have increased globally. Both COVID-19 and mercury exposure present a high prevalence in similar regions: East and Southeast Asia, South America and Sub-Saharan Africa. Since both factors represent a multiorgan threat, a possible synergism could be exacerbating health injuries. Here, we discuss key aspects in mercury intoxication and SARS-CoV-2 infection, describing the similarities shared in clinical manifestations (especially neurological and cardiovascular outcomes), molecular mechanisms (with a hypothesis in the renin-angiotensin system) and genetic susceptibility (mainly by apolipoprotein E, paraoxonase 1 and glutathione family genes). Literature gaps on epidemiological data are also highlighted, considering the coincident prevalence. Furthermore, based on the most recent evidence, we justify and propose a case study of the vulnerable populations of the Brazilian Amazon. An understanding of the possible adverse synergism between these two factors is crucial and urgent for developing future strategies for reducing disparities between developed and underdeveloped/developing countries and the proper management of their vulnerable populations, particularly considering the long-term sequelae of COVID-19.

## 1. COVID-19 Pandemic: A Global Threat

Since its beginning, the ongoing COVID-19 pandemic has caused about 6.81 mi deaths as of 31 January 2023 [1]. Currently, in the United States, approximately 300–500 daily deaths are being reported [2]. In the new COVID-19 reality, each country needs to face the consequences and deal with the pandemic injuries in the social, economic and health sectors, among others. Unfortunately, socio-economic disparities have been evidenced in this context [3], vulnerable people (including racial minorities, refugees and rural communities, among others) being disproportionally affected by this virus [4,5].

COVID-19 is an infectious disease caused by the Severe Acute Respiratory Syndrome Coronavirus 2 (SARS-CoV-2). In the acute phase of COVID-19, a wide range of symptoms are reported, including fever, headache, dyspnea, dry cough, fatigue and smell or taste alterations, among others [6].

During the ongoing pandemic, several risk factors for COVID-19 progression and severity have been described, including medical conditions (such as overweight and obesity [7] and diabetes [8]), lifestyle (such as physical inactivity [9] and tobacco use [10]) and environmental pollution [11,12]. Toxic metal exposure has emerged as an important factor that could modulate the COVID-19 severity risk, thus contributing to worsening the prognosis of patients [11,13]. 

After the acute disease, persistent effects can remain 4 to 12 weeks after infection, characterizing long COVID-19 (also known as the post-acute sequelae of COVID-19, post-COVID-19 condition or post-acute COVID-19 syndrome, among others) [14]. Indeed, the burden of the post-acute sequelae of SARS-CoV-2 infection was estimated in 7–10%, meaning at least 65 million people worldwide [14,15,16]. COVID-19 survivors can develop a number of different sequelae, leading to a decline in quality of life and the need for multidisciplinary care [17].

Respiratory, neurological and neuropsychiatric sequelae of COVID-19 are among the most documented and discussed injuries nowadays [18,19,20]. Although many gaps have yet to be filled in the understanding of the neurological manifestations of long COVID-19, a high frequency of cognition and memory disorders as well as movement disorders have been reported in long-covid patients [20]. Many of these alterations, including both neurodevelopmental and neurodegenerative diseases, have been also related to metal neurotoxicity [21,22], which draws attention to this prominent environmental risk factor and its possible interactions in COVID-19 patients. According to the World Health Organization (WHO), mercury is included in the top 10 chemicals or group of chemicals of major public health concern (https://www.who.int/news-room/fact-sheets/detail/mercury-and-health, accessed on 17 February 2023). Additionally, mercury is ranked third among all the substances considered of major public health concern on the “ATSDR 2019 Substance Priority List” [22]. A total of 140 countries in the world are currently making efforts to reduce mercury emissions to the environment through the Minamata Convention on Mercury (https://www.mercuryconvention.org/en, accessed on 16 February 2023), a global treaty aiming to reduce both environmental mercury pollution as well as human exposure.

## 2. Human Mercury Exposure

Mercury is a toxic metal of natural occurrence. It is the only liquid metal at room temperature, and its properties allow for its use in a wide range of activities and products. For instance, in odontology, this metal is used as dental amalgam; in the cosmetic industry, it is used as an ingredient for skin-lightening products; and in gold mining activity, it is used as the key component separating gold particles from sediments/rocks [23].

Many people consider mercury pollution as a local issue; however, the science has proven that this is far from true. This metal can travel a long distance into the atmosphere, depositing and contaminating areas with no anthropogenic mercury sources, such as the Arctic [24,25]. Therefore, mercury is a global and ubiquitous pollutant, affecting people everywhere. In fact, the United Nations Environment Program (UNEP) has already reported that every person in the world has some amount of mercury in their body due to the different exposure sources such as the intake of contaminated food, the presence of dental amalgam fillings and/or the use of mercury-containing cosmetics products (e.g., skin-lightening products with mercury) [26]. Among all these sources, the chronic exposure to methylmercury (MeHg) via contaminated fish/seafood consumption is the most prevalent and serious one, since organic compounds of mercury are the most toxic forms of the metal. MeHg is considered especially dangerous due to its toxicokinetics properties, including rapid absorption, a wide distribution to all tissues and low elimination [27]. When compared to other mercury species, MeHg has the ability to cross virtually all cell membranes, particularly the blood–placental and blood–brain barriers [27]. Studies have shown human mercury exposure in many rural and urban areas throughout the world, such as the Amazon [23], New York City [28], northern Canada [29] and Muenster city [30], among others.

Once in the human body, this metal is able to cause deleterious effects in multiple systems such as the Central Nervous System (CNS), the respiratory system, the cardiovascular system, the immunological system, the urinary system or the hematological system, among others [31]. Therefore, mercury intoxication is a multiorgan threat, as well as COVID-19. Even at low concentrations, mercury intoxication causes health problems in chronically exposed humans. For instance, the risk of fatal and non-fatal cardiovascular outcomes significantly increases with mercury levels of 1–2 ppm in hair (which reflects the body burden) [32]. This is equivalent to a weekly MeHg intake below the maximum of 1.6 µg/kg currently recommended by the WHO, which results in approximately 2.3 ppm of mercury in hair (for calculations see [27,33]). Thus, even mercury levels below recommendations have already been associated with long-term toxic effects.

Worryingly, the levels of human exposure to mercury during the pandemic could be even higher than those of previous years. It has been confirmed that mercury emissions to the atmosphere have increased in the past decade [34]. Artisanal and small-scale gold mining (ASGM) was responsible for about 38% of the global total mercury emissions [34]. In 2015, 1220 tons of mercury were introduced into the terrestrial and freshwater environments due to ASGM activities [34]. Although during the pandemic, the decreased industrial activity reduced the emissions of other pollutants, particularly carbon [35], in the case of mercury, some events such as increased illegal gold mining, deforestation and fires in South America (especially in the Amazon) may have contributed to maintaining or even increasing mercury emissions [27].

Furthermore, recent evidence on the possible involvement of mercury in COVID-19 severity due to the respiratory dysfunctions associated with metal exposure has been compiled [11]. Because mercury is able to induce respiratory toxicity (e.g., oxidative stress, inflammation and apoptosis in lung cells) as well as immunotoxicity, this metal may be contributing to the excessive inflammatory and impaired immune response against SARS-CoV-2 infection [11]. Considering that COVID-19 is a multi-organ disease affecting many body systems, including the urinary system, the cardiovascular system and the CNS, which, coincidentally, are also damaged by mercury exposure, it is critical to understand the potential adverse synergism between COVID-19 and mercury intoxication.

Contributing to this knowledge, here, we compile and discuss what we currently know about the mechanisms shared by the two pathological conditions. Moreover, based on the most recent evidence, we justify and propose a case study of the vulnerable populations of the Brazilian Amazon and which mechanisms would be priority to analyze for improving our knowledge on the possible synergism.

## 3. COVID-19 and Mercury Intoxication: Is There Any Adverse Synergism?

**Figure 1:**
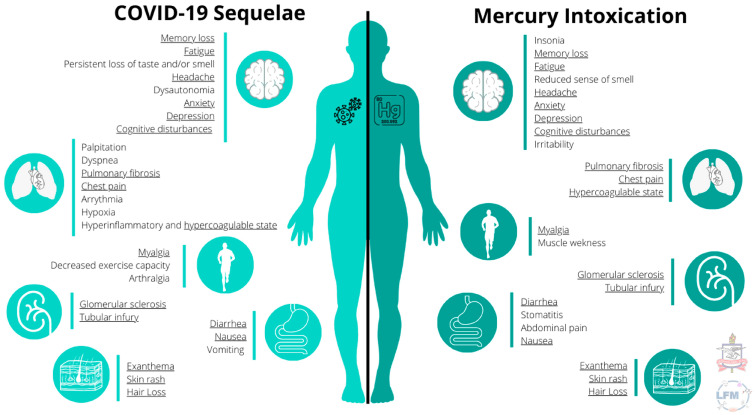
Compiled clinical manifestations described in COVID-19 sequelae (**left**) and mercury intoxication (**right**). The symptoms shared by both conditions are underlined.

Mercury and SARS-CoV-2 infection share many similarities in molecular mechanisms of damage, including but not limited to inflammation, oxidative stress and the disruption of calcium signalization [36,37,38,39]. Thus, we hypothesize that mercury exposure could exacerbate the damage produced by SARS-CoV-2 infection, as both factors influence the same human body systems, share many molecular mechanisms as well as produce similar symptoms such as fatigue, headache, hair loss, anxiety, depression, cognitive disturbances and a hypercoagulable state, among others (Figure 1). 

**Figure 2:**
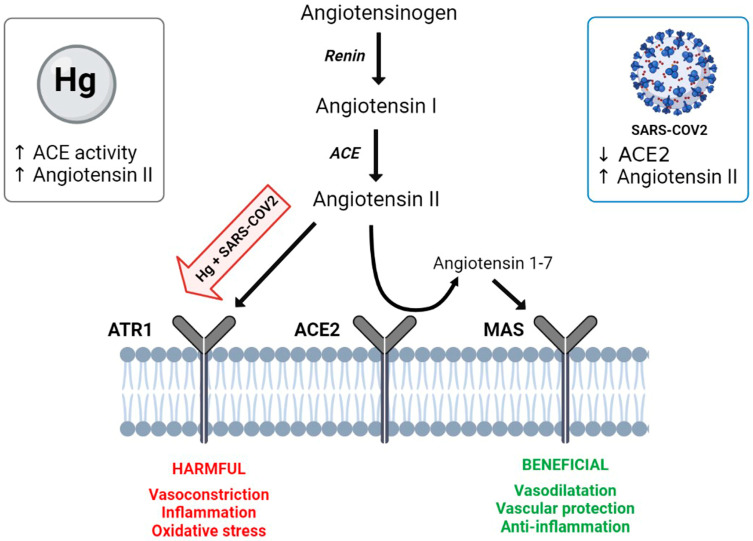
The renin-angiotensin system and the angiotensin-converting enzyme 2 (ACE2)/angiotensin-(1–7)/MAS axis. The angiotensinogen is converted to angiotensin I by renin; then, angiotensin-converting enzyme (ACE) converts angiotensin I to angiotensin II. The binding of angiotensin II to the angiotensin type 1 receptor (AT1R) causes harmful actions, such as vasoconstriction, inflammation and oxidative stress. Angiotensin II can be converted to angiotensin-(1–7) by ACE2. Angiotensin-(1–7) can bind to the MAS receptor to exert benefic actions such as vasodilation, vascular protection and anti-inflammation. SARS-CoV-2 enters cells via ACE2 and, together with the coronavirus, the intact ACE2. Mercury (Hg) intoxication increases the plasma ACE activity. Both insults lead to high plasma levels of angiotensin II. Could SARS-CoV-2 and mercury act synergically to potentiate the harmful effects displayed by angiotensin II? Created with BioRender.com accessed on 6 February 2023.

SARS-CoV-2 enters cells via the angiotensin-converting enzyme 2 (ACE2) receptor, a homolog of angiotensin-converting enzyme (ACE), and during this process, together with the coronavirus, the intact ACE2 or its transmembrane domain is internalized [39,40]. ACE2 is expressed in several different human cells—for instance type II alveolar epithelial cells, myocardial cells, bladder urothelial cells, enterocytes, neurons and glia; therefore, nearly all human organs contain ACE2-expressing cells [40,41]. It is thought that the downregulation of ACE2 might be related to multiple-organ injury in COVID-19 (for a review, see [40]). The ACE2 catalyzes the conversion of angiotensin II to angiotensin-(1–7). The binding of angiotensin II to the angiotensin type 1 receptor (AT1R) causes vasoconstriction, inflammation and oxidative stress, among other things; thus, the ACE2 is able to counteract the negative effects of the renin-angiotensin system via the ACE2/angiotensin-(1–7)/MAS axis (Figure 2) [40]. An expressive increase in plasma angiotensin II levels has been found in COVID-19-positive patients associated with lung injury [42]. Interestingly, mercury intoxication was related to increased blood levels of angiotensin II in both human and animal models [43,44]. Additionally, preclinical evidence has demonstrated that chronic mercury intoxication increases plasma ACE activity, thus producing more angiotensin II (Figure 2) [44]. Given the key role played by ACE in COVID-19 and mercury intoxication (Figure 2), would patients intoxicated with mercury experience a more severe SARS-CoV-2 infection or disease sequelae?

COVID-19 has recently been associated with Kawasaki Disease (KD) in several countries, including France, Italy, the USA and the UK [45]. This disease is an acute systemic vasculitis that affects children, and although its origin is not fully known, it is believed that acute coronavirus infection can cause an exacerbated immune response and manifestation of the disease [46]. Notably, mercury exposure has been long associated with KD [47]. Furthermore, the more severe forms of KD can lead to cardiac complications [48], which are similar to the cardiotoxic effects of mercury in the body, with common outcomes such as acute myocardial infarction, ischemia, thrombosis and myocarditis, among others [49].

Mercury and coronavirus also share pathological effects on cognition [17,23], since the hippocampus and the cerebral cortex are more susceptible to the damage caused by both the metal and the SARS-CoV-2 virus [50]. In both cases, deleterious effects such as a deficit of IQ, memory impairment or verbal comprehension deficits have been described [51]. Such similarities between mercury intoxication and COVID-19 can also be observed by the presence of attention disturbances and the increased risk for neurodegenerative diseases [17,31,51].

Another interesting issue shared, at least in part, by mercury and SARS-CoV-2 virus is the genetic susceptibility. In both cases, recent evidence has pointed to genes such as those codifying for the apolipoprotein E (APOE) [52,53], the paraoxonase 1 (PON1) [54] and the glutathione (GST) family [55]. All these genes have been associated with an increased risk for developing severe COVID-19 as well as for presenting worse outcomes in mercury intoxication. In the latter case, these consequences originate from the genetic modulation of the toxicokinetic (metal distribution, absorption, metabolism and/or excretion) and toxicodynamic (metal/target interactions) processes (reviewed by Arrifano et al. [56]). 

For example, the APOE ε4 allele has been associated with an increased risk of severe COVID-19 as well as post-COVID mental fatigue [52]. In fact, a more than twofold greater mortality rate is reported in patients homozygous for the APOE ε4 allele over those homozygous for the APOE ε3 allele [53]. An increased susceptibility to mercury burden and neurotoxicity has been described in APOE ε4 allele carriers [56,57]. Additionally, the G allele of the rs662 single-nucleotide polymorphism of PON1 is significantly related to COVID-19 severity, increasing the probability of death in homozygous individuals (GG) [54]. The same allele has also been associated with the worse performance of children prenatally exposed to mercury in the intelligence quotient test [58]. Regarding the glutathione family genes, individuals carrying the glutathione S-transferase theta 1 (GSTT1)-/- genotype had a poor survival rate in COVID-19 [55]. Interestingly, the GST polymorphisms are a main influence on the mercury body burden [59]; for instance, the GSTT1-/- genotype is associated with higher mercury levels in the blood of Amazonian riverine people [60].

Considering that our understanding of the genetic susceptibility to the virus infection and its deleterious consequences has just begun, it is likely that the number of genes shown to influence both conditions will increase in the coming years. 

## 4. Mercury Emissions and COVID-19 in the Amazon: A Proposal for a Case Study

Once possible adverse synergisms are hypothesized based on similar molecular mechanisms, it is necessary to test this hypothesis in human populations. To do that, we must look at the most vulnerable populations showing both the highest levels of exposure and a high prevalence of COVID patients. 

Mercury is classically known as the major source of environmental pollution in the Amazonian region, a fact closely related to its wide use in illegal gold mining activity and other anthropogenic interventions such as dams and fires [27,61]. ASGM is the most mercury-emitting activity in the world, being responsible for about twice the emissions from the stationary combustion of coal [34]. Approximately 27% of all global emissions from ASGM originate in the Amazon [62], especially in the Brazilian Amazon, which comprises about 75% of the territory. Furthermore, during the former Brazilian government (2019–2022), the illegal gold mining activity was particularly intensified, and a new gold rush was referred to (https://news.mongabay.com/2022/01/brazils-illegal-gold-rush-is-fueling-corruption-violent-crime-and-deforestation/, accessed on 28 January 2023) (concern has already been raised about the large scale of those activities versus the ASGM definition) [63]). Additionally, the economic challenge imposed by the COVID-19 pandemic pushed thousands of people to work in mining activities in many regions, such as the Amazon and Central Africa [64,65,66]. 

Another important factor contributing to the increase in mercury emissions is the extensive biomass burning and deforestation, which are very recurrent in the Brazilian Amazon (https://www.nytimes.com/2019/12/05/world/americas/amazon-fires-bolsonaro-photos.html, accessed on 27 January 2023). This is because the Amazon forest acts as a “sink” for atmospheric mercury, removing it and fixing it in the tops and leaves of the forest trees as well as in the soil [67]. Fires and deforestation have the potential to release the fixed mercury to the air, contributing to increased mercury emissions. Furthermore, the high mercury levels described in aquatic Amazonian ecosystems are attributed to direct Hg releases (e.g., related to illegal gold mining activity) as well as depositional Hg inputs through rainwater and leakage into the river system in deforested areas, among other factors [68,69]. Additionally, an expressive content of mercury in the Amazonian soil, including in places with no ASGM history, suggests the natural occurrence of this metal [69,70]. Thus, the biomass burning, deforestation and soil alterations can lead to the mercury remobilization and the re-emission of mercury into the air, this being responsible for a substantial amount of global mercury emissions. In fact, the most recent UNEP report reveals that the global mercury emissions from biomass burning are higher than those of geogenic origin such as volcanic emissions [34]. Notably, during the worst period of the pandemic (from January 2020 to January 2021), 8099.02 km^2^ of deforested and burned areas as well as 15,172.73 km^2^ of fire scar areas were recorded in the Brazilian Amazon [71] as a result of the failure to comply with laws, as well as the lack of continuous actions of territorial protection and surveillance. All of these combined actions (biomass burning, deforestation, mining activities) likely increase the current mercury emissions to the atmosphere, with a significant impact on the global emissions, therefore influencing the global human exposure to this metal. 

Considering that the first case of COVID-19 registered in Brazil was on 25 February 2020 [6] and that the first and the second pandemic waves lasted up to mid-2021, at the same time of the worst periods of the pandemic, the Amazon forest was being consumed by fire, deforestation as well as illegal mining activities. In the Brazilian Amazon, COVID-19 caused the collapse of the health and funeral systems. In Manaus City, state of Amazonas, the local politicians refused to follow the public prophylactic policy recommended by the WHO, such as lockdowns, mask use and social distancing [72]; ultimately, Manaus was the Brazilian city with the highest age-adjusted COVID-19 mortality rate (412.5/100,000 people) [73]. In this city, as a result of government denialism [72], hundreds of people died due to running out of oxygen at the hospitals; people literally died of suffocation in hospitals (https://www.reuters.com/business/healthcare-pharmaceuticals/brazils-amazonas-state-running-out-oxygen-covid-19-surges-2021-01-14/, accessed on 30 January 2023). In fact, the high rate of the virus proliferation and transmission was responsible for a new variant of SARS-CoV-2, the gamma or P.1 variant, which emerged in Manaus [74]. 

The high SARS-CoV-2 transmissibility was generally observed in the North of Brazil, which presented a higher rate than other Brazilian regions, particularly in the South, as reported by the Oswaldo Cruz Foundation (Fundação Oswaldo Cruz in Portuguese, also known as FIOCRUZ) (https://portal.fiocruz.br/noticia/estudo-aponta-maior-aceleracao-da-covid-19-em-estados-do-norte-e-nordeste, accessed on 30 January 2023). The accelerated transmission of the coronavirus could be explained in part by the low human development index found in this Brazilian region, which reveals significant inequalities including poverty and a low educational level (which make it difficult to practice the prophylactic recommendations, such as social distancing and buying and wearing masks, among others). Additionally, a significant portion of the Amazonian population lives far from the city centers, hardly having access to healthcare services (which might result in the absence of divulgation and proper guidance on the prophylactic recommendations) [75,76]. 

In addition to the high incidence of SARS-CoV-2 infection, Amazonian populations experience a high exposure to mercury [27,34,77], especially the vulnerable communities, including indigenous, riverine and “*quilombolas*” (afro-descendants living in settlements that preserved their traditional way of life) people. Riverine populations living at the Tapajós River Basin [61,78], in the Tucuruí Lake [77] and at the Madeira River basin [79], as well as indigenous populations such as Munduruku [80] and Yanomami [81], are chronically exposed to high levels of mercury due to the intake of contaminated fish. These traditional Amazonian populations, especially riverine communities, depend on fishing to survive. For these populations, fish is the main protein source of the diet, usually presented in seven or more meals per week, with about 140 g of fish per meal [82,83]. Piscivorous fish are usually contaminated with mercury levels above those legally allowed for human consumption [84]; however, even when fish are supposedly adequate for human consumption, with mercury being below the legal limit, the high frequency of fish intake in these communities means that they are highly exposed, as was easily calculated elsewhere [27].

Unfortunately, Brazil is currently facing a humanitarian crisis: hundreds of adults and children of Yanomami indigenous communities are dying due to starvation, undernutrition, infectious diseases and mercury poisoning (https://www.reuters.com/world/americas/brazil-declares-emergency-over-deaths-yanomami-children-malnutrition-2023-01-22/, accessed on 30 January 2023). Vega et al. found high mercury levels in the hair of Yanomami indigenous people (ranging from 0.4 to 22.1 ppm); the authors also described the presence of several “ASGM camps and boats along the river” in their way to the indigenous village [81]. Recently, Brazilian investigative news has stated that the illegal mining in Yanomami’s territory is currently engaged in by approximately 20,000 miners (or “*garimpeiros*”), who use small and clandestine airplanes to arrive at the mining sites, with more than 40 flights per day (https://www1.folha.uol.com.br/cotidiano/2023/01/garimpo-na-terra-yanomami-nao-se-intimida-com-acao-emergencial-e-40-voos-sao-feitos-por-dia.shtml, accessed on 1 February 2023).

Furthermore, Amazonian populations face several challenges, particularly regarding healthcare; the scenario is even worse considering the traditional Amazonian populations, [85]. The challenges are particularly due to living in remote or rural areas with poor sanitary conditions, low incomes, limited access to healthcare facilities and, frequently, no electricity power, among other things [27,57,76,86].

All the features mentioned previously put Amazonian populations in a vulnerable situation [5,27]. In the COVID-19 era, “vulnerability” was redefined to include not only those individuals considered as biologically more exposed to the risk (such as the elderly, obese people and those with comorbidities) but also all those people who might struggle to cope with the crisis in terms of many aspects, including mental, physical or financial ones [5]. In other words, economically disadvantaged individuals, racial and ethnic minorities, im/migrant populations, lesbian, gay, bisexual, transgender or otherwise queer-identifying (LGBTQ+) populations as well as rural/remote populations who frequently encounter barriers to accessing healthcare services can be considered vulnerable in the new COVID-19 reality [87,88,89]. As recently highlighted, there is therefore a need to develop and implement tailored guidance that meets the specificities of these populations [2], particularly regarding the coronavirus transmission dynamic as well as its relationship with other endemic and chronic diseases and problems such as environmental pollution.

From the point of view of public health, the Amazon has additional factors that make it the most important case study to be carried out to better understand the dynamic of the relationship of COVID-19 and mercury intoxication. For instance, the type of mercury exposure found in many Amazonian populations is the chronic exposure via the consumption of contaminated food (e.g., in the Tapajós area, studies showing high mercury contamination in humans date from the 1990s, meaning at least 30 years of exposure), with many people already presenting signs and symptoms of mercury intoxication [23,27,61,83]. Statistical data on the prevalence of COVID-19 disease and mercury exposure would help us to understand how both factors can be related. Furthermore, the analysis of COVID sequelae and patients in these populations may allow for the investigation of the aspects of COVID-19 in individuals chronically intoxicated with mercury. A wide range of parameters could be evaluated such as neuropsychological outcomes based on questionnaires/clinical evaluation as well as biochemical markers of inflammation and cardiovascular, renal and neuronal injuries using saliva or blood samples’ genetic susceptibility (for a review see [90]). Particularly, studies on genetic susceptibility with Apolipoprotein E and glutathione family genes seem to be especially promising for public health strategies, since previous studies performed in these populations showed a high prevalence for the genotypes associated with a higher risk of COVID-19 [57,60]. 

## 5. Conclusions

An extrinsic factor that supports the urgent necessity of better understanding the COVID-19/mercury intoxication interaction is that both conditions present a high prevalence in the same geographical regions: the regions with the highest emissions of the metal are East and Southeast Asia, South America and Sub-Saharan Africa [34], which share, at the same time, a high circulation of the SARS-CoV-2 virus, a reduced prevalence of vaccination and/or the presence of large groups of vulnerable populations (i.e., racial and ethnic minorities and rural populations, among others). Brazil, for example is responsible for more than 80% of mercury emissions from South America, and at the same time, it has the sad record of being the second-ranked country in the world in terms of the absolute number of COVID-19 deaths and the first-ranked one in terms of the relative number of deaths (https://ourworldindata.org/, accessed on 30 January 2023)

In this perspective, we highlighted the pathological aspects shared by COVID-19 and mercury intoxication, bringing special attention to the potential adverse interaction between these two factors. Therefore, future mechanistic studies at the pre-clinical level addressing molecular and cellular pathways are necessary to better understand this interaction. Additionally, it is essential to perform further studies at the epidemiological level on the association of COVID-19 and mercury intoxication to urgently answer important questions, such as: (1) Is mercury exposure a confirmed risk factor for SARS-CoV-2 virus infection? (2) To what extent will the COVID-19 sequelae in highly exposed individuals be more severe than that in individuals with a lower mercury exposition? (3) Would highly exposed individuals need a different healthcare approach to manage COVID-19 sequelae? (4) What public health strategies would be necessary in the post-pandemic world to simultaneously manage the virus circulation and the exposure of vulnerable populations?

The understanding of the potential adverse synergism between mercury intoxication and COVID-19 infection is crucial and urgent for developing future strategies of public health especially in underdeveloped/developing countries and the proper management of their vulnerable populations, particularly considering the long-term sequelae of COVID-19, which drastically impacts people´s lives.

## Data Availability

Not applicable.
